# Study of Valproic Acid-Enhanced Hepatocyte Steatosis

**DOI:** 10.1155/2016/9576503

**Published:** 2016-02-29

**Authors:** Renin Chang, Mei-Chia Chou, Li-Ying Hung, Mu-En Wang, Meng-Chieh Hsu, Chih-Hsien Chiu

**Affiliations:** ^1^Department of Emergency Medicine, Kaohsiung Veterans General Hospital, No. 386, Dazhong 1st Road, Zuoying District, Kaohsiung 813, Taiwan; ^2^Department of Physical Medicine and Rehabilitation, Kaohsiung Veterans General Hospital, Pingtung Branch, No. 1, Anpin 1st Lane, Jhaosheng Road, Longtan Village, Neipu Township, Pingtung County 912, Taiwan; ^3^Department of Animal Science and Technology, National Taiwan University, No. 50, Lane 155, Sec. 3, Keelung Road, Taipei 106, Taiwan

## Abstract

Valproic acid (VPA) is one of the most widely used antiepilepsy drugs. However, several side effects, including weight gain and fatty liver, have been reported in patients following VPA treatment. In this study, we explored the molecular mechanisms of VPA-induced hepatic steatosis using FL83B cell line-based* in vitro* model. Using fluorescent lipid staining technique, we found that VPA enhanced oleic acid- (OLA-) induced lipid accumulation in a dose-dependent manner in hepatocytes; this may be due to upregulated lipid uptake, triacylglycerol (TAG) synthesis, and lipid droplet formation. Real-time PCR results showed that, following VPA treatment, the expression levels of genes encoding cluster of differentiation 36 (*Cd36*), low-density lipoprotein receptor-related protein 1 (*Lrp1*), diacylglycerol acyltransferase 2 (*Dgat2*), and perilipin 2 (*Plin2*) were increased, that of carnitine palmitoyltransferase I* a *(*Cpt1a*) was not affected, and those of acetyl-Co A carboxylase *α* (*Acca*) and fatty acid synthase (*Fasn*) were decreased. Furthermore, using immunofluorescence staining and flow cytometry analyses, we found that VPA also induced peroxisome proliferator-activated receptor *γ* (PPAR*γ*) nuclear translocation and increased levels of cell-surface CD36. Based on these results, we propose that VPA may enhance OLA-induced hepatocyte steatosis through the upregulation of PPAR*γ*- and CD36-dependent lipid uptake, TAG synthesis, and lipid droplet formation.

## 1. Introduction

Valproic acid (2-propylpentanoic acid, VPA), a chemically synthesized small compound, has been one of the most widely used antiepilepsy drugs over the past 40 years. It is also commonly prescribed for patients with bipolar disorder, neuropathic pain, migraine headache, and schizophrenia. However, clinical follow-up studies have reported that more than 40% of patients who received VPA also developed unexpected obesity and fatty liver disease [1–3]. Previous studies have proposed the involvement of downregulated mitochondrial *β*-oxidation, accumulation of VPA metabolites (4-ene-VPA and 2,4-diene-VPA), and carnitine deprivation in VPA-induced liver injury [4, 5]. Furthermore, animal studies have demonstrated that VPA alters hepatic triacylglycerol (TAG) and cholesterol biosynthesis, fatty acid catabolism, and lipid transport-related gene expression patterns [6, 7]. However, how VPA affects hepatic lipid metabolism to induce fatty liver remains largely unknown.

Hepatic steatosis, also known as fatty liver, may be caused by imbalanced lipid metabolism, including lipid uptake/secretion,* de novo* lipogenesis, and fatty acid oxidation [8]. According to previous studies, of the TAG stored in steatotic hepatocytes, 59% were formed from circulating free fatty acids, 26% from* de novo* lipogenesis, and 15% from dietary chylomicron remnants [9, 10]. These data emphasize the importance of lipid transport regulation on fatty liver development and provide critical clues regarding VPA-induced hepatic steatosis. Although it is believed that fatty acids are able to enter cells via passive diffusion, carrier-mediated fatty acid uptake has also been proposed previously [11, 12]. Fatty acid translocase (FAT)/CD36 is a membrane-associated multifunctional receptor which has two transmembrane segments and an extracellular glycosylated loop. CD36 regulates lipid uptake in different cells via binding to different ligands including long-chain fatty acids [13], and native/oxidized lipoproteins [14–16]. Importantly, a recent study showed that increased hepatic CD36 expression is highly correlated with human insulin resistance and nonalcoholic steatohepatitis (NASH) development [17].

On the other hand, the nuclear transcription factor peroxisome proliferator-activated receptor *γ* (PPAR*γ*), which is a pivotal regulator of lipid metabolism, may also play a critical role in VPA-induced fatty liver disease. PPAR*γ*, along with the liver X receptor (LXR) and pregnane X receptor (PXR), was found to be an upstream regulator of CD36; the presence of PPAR*γ*, LXR, and PXR binding sites in the* Cd36* promoter was previously identified [18]. In addition, PPAR*γ* was also demonstrated to be a major mediator of oleic acid- (OLA-) induced hepatic lipogenesis and lipid droplet formation [19] and found to be highly expressed in high-fat diet-fed mice [20]. Based on these studies, we investigated the involvement of PPAR*γ*- and CD36-regulated lipid metabolism in VPA-induced hepatic steatosis.

## 2. Materials and Methods

### 2.1. Chemicals

Valproic acid sodium salt, Nile Red, 3-(4,5-Dimethyl-2-thiazolyl)-2,5-diphenyl tetrazolium bromide (MTT), F12K medium powder, Hoechst 33342, dimethyl sulfoxide (DMSO), and sodium oleate were purchased from Sigma-Aldrich. Fatty acid-free bovine serum albumin was purchased from Merck.

### 2.2. Cell Culture

FL83B cells were obtained from the Taiwan Bioresource Collection and Research Center (BCRC number: 60325) and cultured in F12K medium supplemented with 10% fetal bovine serum and 1% penicillin/streptomycin (Gibco) at 37°C in a humidified atmosphere with 5% CO_2_. To make the stock medium containing 4 mM OLA, sodium oleate was conjugated to BSA at a molar ratio of 6 : 1 in F12K medium. For VPA stock preparation, VPA was dissolved in distilled water. All compounds were sterilized by filtration.

### 2.3. Intracellular Lipid Staining and Quantification

To determine intracellular lipid accumulation, a Nile Red and Hoechst 33342 double staining protocol was applied. Briefly, cells were seeded on either a 0.17 mm coverslip or a black 96-well plate (Nunc) and incubated overnight to allow adherence. After 24-hour OLA and VPA treatment, cells were washed with PBS and then fixed with 4% paraformaldehyde for 10 min. After fixing, cells were washed again with PBS and then stained with Nile Red (1 *μ*g/mL) and Hoechst 33342 (2 *μ*g/mL) in the dark for 30 min. For microscopy imaging, cells were washed with PBS and then mounted with ProLong Gold Antifade Mountant (Molecular Probes). Images were captured using an Olympus IX-70 inverted microscope. For quantitative analyses, stained cells were washed with PBS, and the Nile Red (excitation at 485 nm, emission at 535 nm) and Hoechst 33342 (excitation at 350 nm, emission at 461 nm) fluorescent signals were quantified using a Biotek H1 Hybrid Microplate Reader.

### 2.4. Cell Viability Assay

The effects of OLA and VPA on cell viability changes were analyzed by the MTT assay. Briefly, cells were seeded in 96-well plates (Nunc) and allowed to adhere overnight before treatment. After 24-hour treatment, the medium was replaced with fresh medium containing MTT (5 mg/mL dissolved in PBS for stock solution, diluted 1 : 10 with normal medium for assay). After a 1.5-hour incubation at 37°C, the MTT solution was removed, and then 200 *μ*L of DMSO was added to each well to dissolve the insoluble formazan. Cell viability was determined by measuring OD values at 570 nm (reference at 690 nm) using a BioTek *μ*Quant microplate spectrophotometer.

### 2.5. qPCR Analyses

For qPCR analyses, total RNA were extracted from cells using the TRIzol Reagent (Invitrogen) following the recommended protocol provided by the manufacturer. After total RNA extraction, first-strand cDNA was synthesized using the Tetro Reverse Transcription kit (Bioline) with a mixture of oligo (dT)_20_ and random hexamer primers. cDNA samples were then quantified and diluted into the same concentrations for subsequent qPCR assays. To determine mRNA expression changes, the relative quantification (ΔΔC_T_) method was performed using the StepOne Real-Time PCR System with Fast SYBR Green Master Mix reagent (Applied Biosystems) and custom-designed gene-specific primer pairs (Table 1). In our study, all gene expression levels were normalized with that of 18S rRNA as the internal control.

### 2.6. Flow Cytometry

To investigate the effects of VPA on cell-surface CD36 expression levels, flow cytometry analyses were conducted. Treated cells on 10 cm dishes (Nunc) were harvested by trypsinization and centrifugation. Cells were washed in ice-cold PBS and resuspended in FACS buffer (2% FBS and 0.1% NaN_3_ in PBS). Mouse IgA isotype control antibody (GTX35045, GeneTex) or anti-CD36 mouse monoclonal IgA antibody (sc-13572, Santa Cruz Biotechnology) was added and incubated on ice for 30 min. After primary antibody labeling, cells were washed once with FACS buffer and then incubated with DyLight 650-conjugated goat-anti-mouse IgA antibody (Abcam) in the dark for another 30 min on ice. After secondary antibody labeling, cells were washed again in FACS buffer, filtered with Cell Strainer Cap (BD Biosciences), and then analyzed on LSRFortessa cell analyzer (BD Biosciences) with BD FACSDiva software.

### 2.7. Western Blotting

For Western blotting, protein from cultured FL83B cells was extracted using RIPA buffer, followed by BCA protein (Pierce) quantification. Samples were then mixed with Laemmli buffer and boiled at 95°C for 10 min. Denatured protein samples were then separated by SDS-PAGE and transferred to PVDF membranes (Bio-Rad) using standard protocol. After transfer, membranes were incubated with specific antibodies at 4°C overnight, then washed with TBST, and incubated with HRP conjugated secondary antibodies for 1 hour at room temperature. For signal developing, ECL select reagent (GE healthcare) and ChemiDoc*™* Touch Imaging System (Bio-Rad) were used. Densitometry analyses were performed using Image J software (National Institutes of Health).

### 2.8. Immunofluorescence Staining

In this study, an immunofluorescence staining technique was conducted to investigate the PPAR*γ* nuclear translocation state. Briefly, cells were seeded, treated, and fixed as mentioned in the lipid staining section and then permeabilized with 0.25% Triton X-100 in PBS for 2 min at room temperature. After permeabilization, cells were washed in PBS, blocked with 1% BSA and 0.3 M glycine in PBS for 30 min at room temperature, and then stained with anti-PPAR*γ* antibody (H-100, Santa Cruz Biotechnology) overnight at 4°C. On the next day, cells were washed three times with PBS for 5 min each to remove excessive primary antibodies. After the wash, Hoechst 33342- (2 *μ*g/mL) and DyLight 488-conjugated goat-anti-rabbit IgG antibodies (Jackson ImmunoResearch Laboratories) were added and incubated in the dark for 1 hour at RT. After fluorescent labeling, slides were washed, mounted, and imaged under an Olympus IX-70 microscope as described before.

### 2.9. Statistical Analysis

Statistical analyses were conducted using SigmaPlot software (Version 12.0, Systat Software). All values were expressed as means ± SEM (the number of biological replicates in each experiment is indicated in the figure legend). The fluorescence intensity and mRNA/protein expression data were analyzed by one-way and two-way ANOVA, followed by Duncan's multiple comparison. *P* < 0.05 was considered a significant difference.

## 3. Results

### 3.1. VPA Enhances Oleic Acid-Induced Lipid Accumulation and Causes Lipotoxicity in FL83B Cells

**Figure 1:**
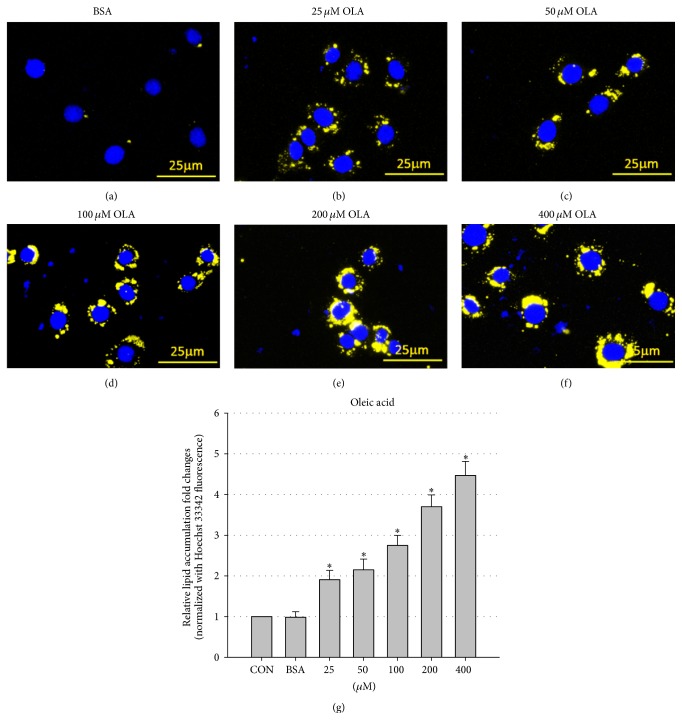
OLA induces lipid accumulation in FL83B cells in a dose-dependent manner. Microscopic images of FL83B cells treated with BSA only (a), or 25 *μ*M (b), 50 *μ*M (c), 100 *μ*M (d), 200 *μ*M (e), and 400 *μ*M (f) BSA-conjugated OLA for 24 hours. Cells were fixed and stained with Hoechst 33342 (blue, nucleus) and Nile Red (yellow, intracellular lipid droplets). Quantification of intracellular lipid accumulation was performed using a fluorescent microplate reader (g). Values are means ± SEM of four independent experiments. *∗* above the bars refer to significant differences (*P* < 0.05). CON: control, treated with normal culture medium only.

**Figure 2:**
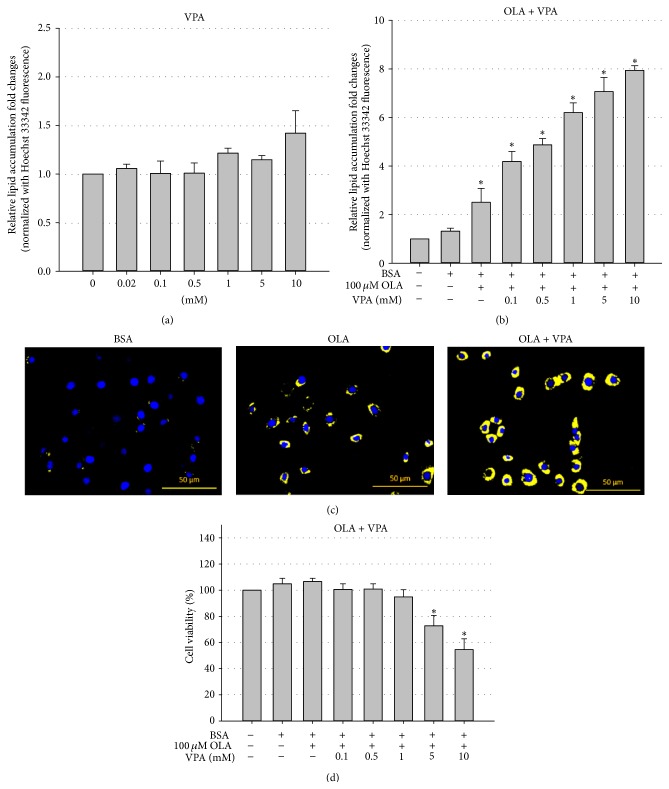
VPA enhances OLA-induced lipid accumulation in FL83B cells and induces cytotoxicity. Nile Red and Hoechst 33342 double staining showed that 24-hour VPA single treatment (a) did not significantly induce steatosis but enhanced OLA- (100 *μ*M) induced intracellular neutral lipid accumulation (b) and lipid droplet formation (OLA = 100 *μ*M, VPA = 1 mM) (c) in a dose-dependent manner in FL83B cells. MTT cell viability results also indicate high doses (5 and 10 mM) of VPA-induced lipotoxicity in FL83B cells (d). Values are means ± SEM of four independent experiments. *∗* above the bars refer to significant differences (*P* < 0.05).

To address the effects of VPA on hepatic steatosis, we developed an* in vitro* hepatic steatosis model using the mouse FL83B cell line and a Nile Red lipid staining technique. FL83B is a noncancer hepatocyte cell line originated from fetal C57BL/6 mice [23], the most widely used strain for studies of fatty liver, obesity, and many other metabolic diseases. Using Nile Red and Hoechst 33342 double staining (Nile Red stains intracellular neutral lipids, while Hoechst 33342 stains the nucleus for cell number normalization), we found that 24-hour OLA treatment can induce lipid droplet formation and neutral lipid accumulation in a dose-dependent manner in FL83B cells (Figure 1). Next, we evaluated the steatosis-promoting effects of VPA using the same staining method. Our results showed that although treatment with VPA alone did not induce significant lipid accumulation in FL83B cells (Figure 2(a)), VPA enhanced 100 *μ*M OLA-induced steatosis in a dose-dependent manner (tested doses ranged from 0.1 to 10 mM) in FL83B cells (Figures 2(b) and 2(c)). More importantly, we demonstrated that high-dose (5 and 10 mM) VPA single (data not shown) or cotreatment also induces significant cytotoxicity (Figure 2(d)), which may contribute to NASH development.

### 3.2. Effects of VPA on the Expression of Lipid Metabolic Genes

**Figure 3:**
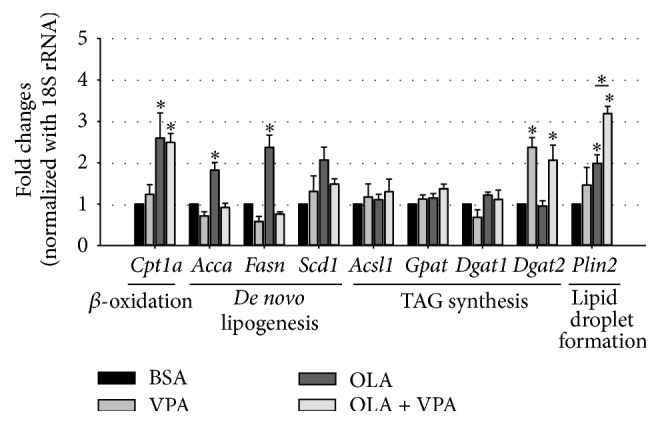
Regulation of expression of lipid metabolism-related genes by OLA and VPA. The mRNA levels of lipid catabolism/anabolism-related genes including* Cpt1*,* Acca*,* Fasn*,* Scd1*,* Acsl1*,* Gpat*,* Dgat1*,* Dgat2*, and* Plin2* in FL83B cells treated with BSA, 100 *μ*M OLA, 1 mM VPA, or 100 *μ*M OLA plus 1 mM VPA for 24 hours were analyzed using real-time PCR. Relative fold changes were calculated using Ct values obtained from three independent experiments and are shown as means ± SEM. *∗* above the bars refer to significant differences (*P* < 0.05).

In order to investigate the molecular mechanisms of VPA-enhanced hepatic steatosis, we assayed the expression patterns of lipid metabolic genes using real-time PCR. Our results suggest that VPA enhances hepatic steatosis possibly by increasing TAG synthesis and lipid droplet formation, but not by upregulating* de novo* lipogenesis or decreasing fatty acid oxidation. As shown in Figure 3, VPA cotreatment did not inhibit the OLA-induced expression of* Cpt1a*, which encodes the rate-limiting enzyme carnitine palmitoyltransferase I, controlling hepatic mitochondrial *β*-oxidation. Furthermore, we found that VPA did not increase but rather decreased the expression of* de novo* lipogenesis genes including* Acca* (acetyl-CoA carboxylase *α*, catalyzing the conversion of malonyl-CoA from acetyl-CoA, the rate-limiting step of fatty acid synthesis),* Fasn* (fatty acid synthase, synthesizing palmitic acid using acetyl-CoA and malonyl-CoA as substrates), and* Scd1* (stearoyl-CoA desaturase-1, the key enzyme involved in monounsaturated fatty acid production via introducing double bonds into palmitoyl-CoA and stearoyl-CoA).

However, when assaying TAG synthesis genes including* Acsl1* (acyl-CoA synthetase long-chain family member 1),* Gpat* (glycerol-3-phosphate acyltransferase),* Dgat1* (diacylglycerol acyltransferase 1), and* Dgat2* (diacylglycerol acyltransferase 2), we found significantly increased* Dgat2* expression following treatment with VPA alone and in combination with OLA (Figure 3). In addition, we found that VPA also enhanced the expression of* Plin2* (perilipin 2), which encodes a lipid droplet membrane-associated protein (Figure 3). These data suggest that VPA may enhance OLA-induced hepatic steatosis by increasing TAG synthesis and lipid droplet formation.

### 3.3. Effects of VPA on Lipid Transport Genes

**Figure 4:**
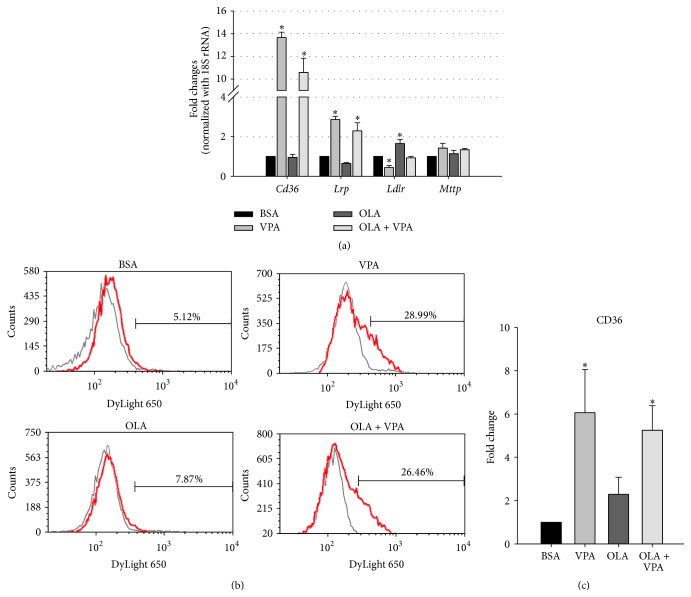
VPA increases the mRNA and cell-surface protein expression levels of fatty acid translocase CD36. The mRNA expression levels of lipid transport-related genes including* Cd36*,* Lrp*,* Ldlr*, and* Mttp* were analyzed using real-time PCR (a). Relative fold changes were calculated using Ct values obtained from three independent experiments and are shown as means ± SEM. *∗* above the bars refer to significant differences (*P* < 0.05). The Fl83B cell-surface CD36 expression levels were analyzed by flow cytometry using mouse anti-CD36 antibody and DyLight 650-conjugated secondary antibody, and representative histograms (b) and quantitative flow cytometry data (c) are shown. *∗* above the bars refer to significant differences (*P* < 0.05). For background fluorescent subtraction, mouse isotype IgG antibody was used (OLA = 100 *μ*M, VPA = 1 mM, 24 hours).

In addition to lipid anabolic and catabolic genes, we also evaluated the regulation of lipid transport genes using real-time PCR. Among the four genes we assayed including* Cd36* (cluster of differentiation 36),* Lrp1* (low-density lipoprotein receptor-related protein 1),* Ldlr* (low-density lipoprotein receptor), and* Mttp* (microsomal triacylglycerol transfer protein), we found that VPA treatment profoundly increased the mRNA levels of* Cd36* and* Lrp*, which facilitate the import of long-chain fatty acids and VLDL (very low-density lipoproteins), respectively (Figure 4(a)). On the other hand, another VLDL uptake receptor gene,* Ldlr,* showed slightly decreased expression, and the lipid export protein gene* Mttp* remained unchanged under VPA treatment (Figure 4(a)). These data suggest the involvement of increased lipid uptake in VPA-enhanced hepatic steatosis, especially CD36-mediated fatty acid uptake, because its mRNA level was extraordinarily upregulated.

To further confirm the importance of CD36 in VPA-induced hepatic steatosis, we analyzed cell-surface CD36 levels using flow cytometry analyses. Consistent with the real-time PCR results, we found that cell-surface CD36 levels were significantly increased under VPA treatment (Figures 4(b) and 4(c)), indicating that VPA may increase lipid uptake in FL83B cells by elevating CD36 expression and translocation to the cell membrane.

### 3.4. VPA Increased OA Induced PPAR*γ* Protein Expression and Nuclear Translocation

**Figure 5:**
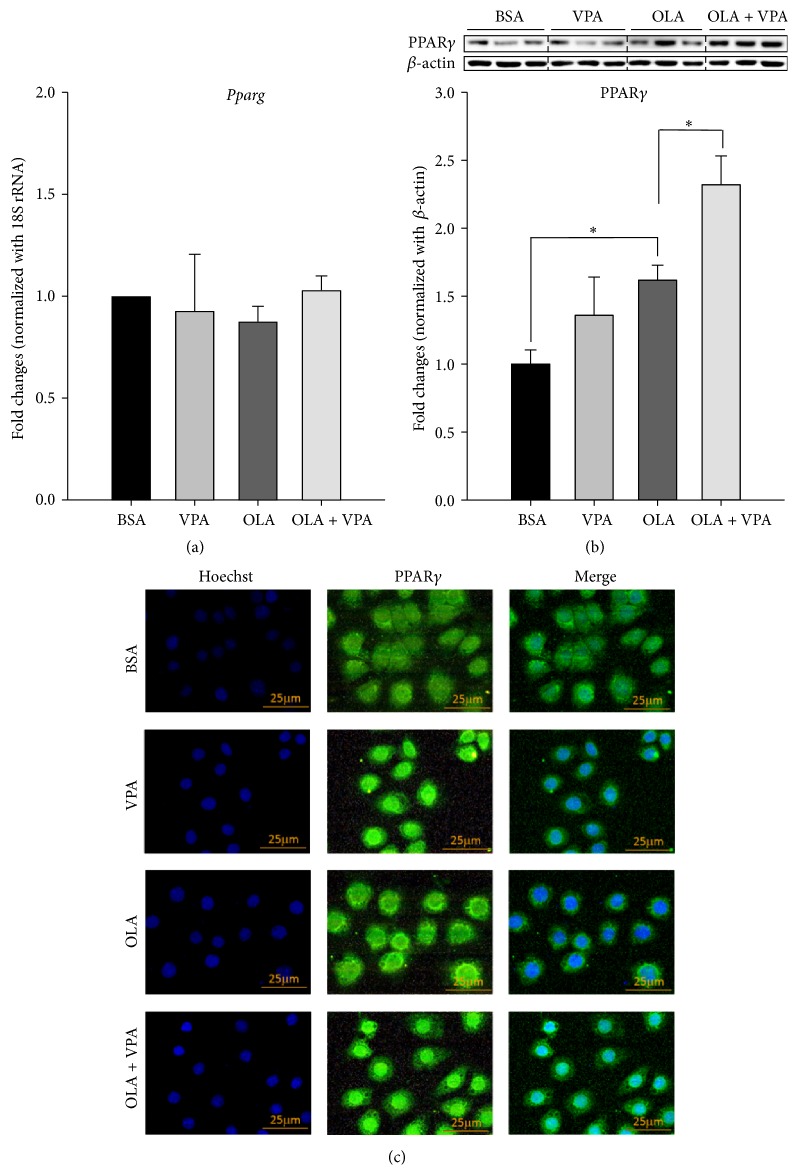
VPA enhances oleic acids increased PPAR*γ* protein expression and nuclear translocation, but not the mRNA levels. Real-time PCR (a), Western blotting (b), and immunofluorescence staining (c) were conducted following 24-hour treatment with BSA, VPA (1 mM), OLA (100 *μ*M), or OLA plus VPA. For real-time PCR, relative fold changes were calculated using Ct values obtained from three independent experiments and are shown as means ± SEM. *∗* above the bars refer to significant differences (*P* < 0.05). Densitometric analyses for Western blotting were conducted for sample sets obtained from three independent experiments, and results are shown as means ± SEM. *∗* above the bars refer to significant differences (*P* < 0.05). In the immunofluorescence staining images, nuclear and intracellular PPAR*γ* proteins were stained by Hoechst 33342- (blue) and DyLight 488-conjugated antibody (green), respectively.

Finally, we also addressed the role of the nuclear transcription factor PPAR*γ* in VPA-induced steatosis. Because PPAR*γ* was previously reported to upregulate the expression of* Plin2*,* Cd36*, and many other steatotic genes in liver [18, 24], we evaluated the mRNA, protein expression levels, and protein nuclear translocation of PPAR*γ* in FL83B cells under VPA treatment. As shown in Figure 5, we found that although the mRNA levels of* Pparg* were not altered by either VPA or OLA treatment (Figure 5(a)), VPA did increase OA induced PPAR*γ* protein expression (Figure 5(b)) and nuclear translocation (Figure 5(c)). Using the immunofluorescence staining technique, we found that VPA on its own triggered and also enhanced OLA-induced PPAR*γ* nuclear translocation. Our data suggests that PPAR*γ* may be an upstream regulator via which VPA induces hepatic steatosis.

## 4. Discussion

VPA is currently one of the most widely used antiepilepsy drugs in the world. However, according to the clinical statistics, a high prevalence of fatty liver diseases induced by unknown mechanisms is observed in VPA-treated patients [1–3]. Therefore, we developed a highly reproducible* in vitro* cell model to investigate the possible mechanisms of VPA-induced hepatic steatosis. In this study, we used FL83B, a noncancer hepatocyte cell line derived from fetal C57BL/6 mice, which presents similar morphology and physiological functions to those of normal hepatocytes [23]. Since FL83B is derived from C57BL/6 mice, the most commonly used strain in studies of obesity, fatty liver, and many other metabolic diseases, this model may easily be used to interpret* in vivo* findings.

To induce steatosis in FL83B cells, we treated the cells with BSA-conjugated OLA and analyzed the steatotic levels using Nile Red and Hoechst 33342 double staining. According to Greenspan et al., when excited by light of wavelength 450–500 nm, the lipophilic fluorescent dye Nile Red incorporated in neutral lipids emits strong yellow-gold fluorescence with wavelengths greater than 528 nm [25]. To further increase the accuracy of Nile Red lipid staining results, we also used Hoechst 33342 nuclear staining in our system for cell number normalization. Via this, we can readily image and quantify intracellular neutral lipid-rich droplets using fluorescent microscopy and a microplate reader, respectively. As shown in Figure 1, we confirmed that this staining method successfully detects OLA-induced lipid accumulation in FL83B cells.

Next, we used this cell model to evaluate whether VPA has direct effects on hepatocyte steatosis. According to the results shown in Figure 2, we found that treatment with VPA alone at doses ranging from 0.02 to 10 mM did not significantly induce steatosis in FL83B cells (Figure 2). However, when cells were cotreated with 100 *μ*M OLA, VPA significantly enhanced OLA-induced lipid accumulation in a dose-dependent manner (Figures 2(b) and 2(c)). More importantly, we found that VPA not only enhanced fatty acid-induced hepatocyte steatosis, but also caused cell death (Figure 2(c)). These results are consistent with previous* in vivo* and clinical findings: a study by Zhang et al. revealed that low-dose VPA treatment did not induce profound hepatic steatosis and liver injury in rats. However when rats were fed a high-fat diet for 8 weeks, VPA cotreatment significantly increased hepatic steatosis and liver cell death [26]. Interestingly, although VPA treatment* per se* was found to induce body weight gain and fatty liver, clinical data also revealed that VPA-induced fatty liver was more significant in patients with higher body weight [2].

Since VPA only enhanced OLA-induced lipid accumulation in FL83B cells but did not induce steatosis in the absence of OLA, we hypothesized that VPA may induce hepatic steatosis via increasing lipid uptake, neutral lipid synthesis, and lipid droplet formation, rather than by downregulating beta-oxidation or upregulating* de novo* lipogenesis in hepatocytes. Consistent with this hypothesis, we found that VPA significantly upregulates lipid uptake, TAG synthesis, and lipid droplet formation, but not the expression profiles of genes involved in* de novo* lipogenesis and beta-oxidation. Using real-time PCR, we found that VPA did not alter the mRNA levels of* Cpt1a*. Furthermore, VPA did not increase the mRNA expression of* Scd1* and even decreased the OLA-induced expression of* Acca* and* Fasn* (Figure 3). These data indicate that VPA may not directly induce lipid accumulation in hepatocytes via increasing* de novo* lipogenesis or downregulating beta-oxidation. However, when assaying the mRNA expression levels of* Acsl1*,* Gpat*,* Dgat1,* and* Dgat2*, which encode the key enzymes in TAG synthesis, we found that VPA significantly increased the level of* Dgat2*. Furthermore, we found that VPA also remarkably enhanced OLA-induced PLIN2 mRNA expression, which is important for OLA-induced lipid droplet formation [27]. Besides, it is worth noting here that although VPA treatment alone induced* Dgat2* expression, lipid accumulation only increased when cells were cotreated with OLA, indicating the importance of fatty acid uptake in VPA-induced hepatic steatosis (Figure 3).

Therefore, we next assayed expression changes in lipid uptake/export related genes including* Cd36*,* Lrp*,* Ldlr*, and* Mttp*. As shown in Figure 4(a), we found that although VPA treatment slightly decreased* Ldlr* expression, it significantly increased* Lrp* expression both alone and in combination with OLA treatment. The decrease in* Ldlr* expression here may be compensated by the increased* Lrp* expression, because these molecules were found to mediate the intake of similar lipoproteins into liver [28]. On the other hand, no changes were observed in the expression of the TAG export protein gene* Mttp* in all treatment groups. Interestingly, we found that VPA significantly increased the mRNA expression of CD36 more than 10-fold, both alone and in combination with OLA treatment. CD36 is a cell membrane-bound protein, which plays an important role in the cellular uptake of long-chain fatty acids, native lipoproteins, and oxidized LDL [15, 21, 29, 30]. Previous studies have shown decreased liver TAG content and increased plasma free fatty acid levels in CD36 knockout mice, indicating the significance of CD36 in the control of hepatic lipid transportation balance [31]. More importantly, using a flow cytometry assay, we found that VPA not only increased CD36 mRNA levels, but also enhanced cell-surface CD36 protein expression (Figures 4(b) and 4(c)).

Because the expression of CD36, DGAT2, and PLIN2 in liver is previously known to be regulated by PPAR*γ* [18, 32, 33], a crucial nuclear transcription factor controlling cellular glucose and lipid metabolism, we also investigated whether PPAR*γ* is involved in VPA-induced hepatic steatosis. Upon ligand binding and activation, PPAR*γ* translocates to the nucleus, forms heterodimers with the retinoid X receptor (RXR), and binds to DNA peroxisome proliferator hormone response element (PPRE) regions to regulate downstream gene expression levels. PPAR*γ* is highly expressed in adipocytes, acting as an important regulator of adipogenesis [34]. Although its expression level in liver is lower than in adipose tissue, previous studies have shown that PPAR*γ* regulates hepatic lipid accumulation; high-fat diet-fed mice exhibited significant elevation of PPAR*γ* expression [20, 24, 35, 36]. Our real-time PCR, Western blotting, and immunofluorescence staining results revealed that VPA enhanced OLA increased PPAR*γ* protein expression and nuclear translocation, but not the mRNA levels (Figure 5). Importantly, these data also indicated that the regulating effects of VPA on PPAR*γ* may rely on translation or protein degradation regulations, instead of transcriptional modulations.

## 5. Conclusions

In conclusion, our data indicate that VPA may enhance hepatic steatosis via increasing the protein expression and transcriptional activity of PPAR*γ*, as well as by inducing the expression of downstream genes including* Cd36*,* Dgat2*, and* Plin2* to increase lipid uptake, TAG synthesis, and lipid droplet formation in hepatocytes.

## Figures and Tables

**Table 1:** Sequences of primer pairs used in study.

Gene	Forward/reverse primer sequences (5′ to 3′)	Reference
18S rRNA	CGGACAGGATTGACAGATTG CAAATCGCTCCACCAACTAA	NT039649

*Cpt1a*	CTGAGCCATGAAGCCCTCAA CACACCCACCACCACGATAA	NM013495

*Acca*	GGAGCTAAACCAGCACTCCC GGCCAAACCATCCTGTAAGC	NM133360

*Fasn*	TAGAGCAGGACAAGCCCAAG GAGGCGTAGTAGACAGTGCAGAG	NM007988

*Scd1*	GGCCTGTACGGGATCATACTG CAGAGCGCTGGTCATGTAGTA	NM009127

*Acsl1*	AGCACCGTACACTGGAGGAA AGGAAAACCTCTGGTCCACTG	NM007981

*Gpat*	ATGAAACGCACACAAGGCAC CCCTTATGGACGTCTCGCTC	NM008149

*Dgat1*	TTGACCTCAGCCTTCTTC CATTGCCATAGTTCCCTTG	NM010046

*Dgat2*	GCTGGCATTTGACTGGAACA GCCACACGGCCCAGTTT	NM026384

*Plin2*	GAACAGTGGAGTAGATAATG TGAGAGCCTGGTGATAAG	NM007408

*Cd36*	TTACACATACAGAGTTCGTTATC TCCAACAGACAGTGAAGG	NM001159558

*Lrp*	GACCGACTGGCGAACAAAT CTGGGTGTTGGTCCTCTGTA	Degrace et al. [21]

*Ldlr*	CTGTGGGCTCCATAGGCTATCT GCGGTCCAGGGTCATCTTC	Lelliott et al. [22]

*Mttp*	ATTGAGCGGTCTGGATTTACAAC AGGTAGTGACAGATGTGGCTTTTG	Lelliott et al. [22]

*Pparg*	CGGGCTGAGGAGAAGTCACA GTCTGTCACACAGTCCTGTCA	NM001127330

## References

[1] Saleh D. A. A., Ismail M. A., Ibrahim A. M. (2012). Non alcoholic fatty liver disease, insulin resistance, dyslipidemia and atherogenic ratios in epileptic children and adolescents on long term antiepileptic drug therapy. *Pakistan Journal of Biological Sciences*.

[2] Luef G. J., Waldmann M., Sturm W. (2004). Valproate therapy and nonalcoholic fatty liver disease. *Annals of Neurology*.

[3] Verrotti A., Di Marco G., La Torre R., Pelliccia P., Chiarelli F. (2009). Nonalcoholic fatty liver disease during valproate therapy. *European Journal of Pediatrics*.

[4] Knapp A. C., Todesco L., Beier K. (2008). Toxicity of valproic acid in mice with decreased plasma and tissue carnitine stores. *Journal of Pharmacology and Experimental Therapeutics*.

[5] Peterson G. M., Naunton M. (2005). Valproate: a simple chemical with so much to offer. *Journal of Clinical Pharmacy and Therapeutics*.

[6] Lee M.-H., Hong I., Kim M. (2007). Gene expression profiles of murine fatty liver induced by the administration of valproic acid. *Toxicology and Applied Pharmacology*.

[7] Lee M.-H., Kim M., Lee B.-H. (2008). Subchronic effects of valproic acid on gene expression profiles for lipid metabolism in mouse liver. *Toxicology and Applied Pharmacology*.

[8] Canbay A., Bechmann L., Gerken G. (2007). Lipid metabolism in the liver. *Zeitschrift für Gastroenterologie*.

[9] Diraison F., Moulin P. H., Beylot M. (2003). Contribution of hepatic de novo lipogenesis and reesterification of plasma non esterified fatty acids to plasma triglyceride synthesis during non-alcoholic fatty liver disease. *Diabetes and Metabolism*.

[10] Donnelly K. L., Smith C. I., Schwarzenberg S. J., Jessurun J., Boldt M. D., Parks E. J. (2005). Sources of fatty acids stored in liver and secreted via lipoproteins in patients with nonalcoholic fatty liver disease. *The Journal of Clinical Investigation*.

[11] Stremmel W., Pohl J., Ring A., Herrmann T. (2001). A new concept of cellular uptake and intracellular trafficking of long-chain fatty acids. *Lipids*.

[12] Ehehalt R., Füllekrug J., Pohl J., Ring A., Herrmann T., Stremmel W. (2006). Translocation of long chain fatty acids across the plasma membrane—lipid rafts and fatty acid transport proteins. *Molecular and Cellular Biochemistry*.

[13] Baillie A. G. S., Coburn C. T., Abumrad N. A. (1996). Reversible binding of long-chain fatty acids to purified FAT, the adipose CD36 homolog. *Journal of Membrane Biology*.

[14] Calvo D., Gómez-Coronado D., Suárez Y., Lasunción M. A., Vega M. A. (1998). Human CD36 is a high affinity receptor for the native lipoproteins HDL, LDL, and VLDL. *Journal of Lipid Research*.

[15] Endemann G., Stanton L. W., Madden K. S., Bryant C. M., White R. T., Protter A. A. (1993). CD36 is a receptor for oxidized low density lipoprotein. *Journal of Biological Chemistry*.

[16] Nicholson A. C., Frieda S., Pearce A., Silverstein R. L. (1995). Oxidized LDL binds to CD36 on human monocyte-derived macrophages and transfected cell lines. Evidence implicating the lipid moiety of the lipoprotein as the binding site. *Arteriosclerosis, Thrombosis, and Vascular Biology*.

[17] Miquilena-Colina M. E., Lima-Cabello E., Sánchez-Campos S. (2011). Hepatic fatty acid translocase CD36 upregulation is associated with insulin resistance, hyperinsulinaemia and increased steatosis in non-alcoholic steatohepatitis and chronic hepatitis C. *Gut*.

[18] Zhou J., Febbraio M., Wada T. (2008). Hepatic fatty acid transporter Cd36 is a common target of LXR, PXR, and PPAR*γ* in promoting steatosis. *Gastroenterology*.

[19] Morán-Salvador E., López-Parra M., García-Alonso V. (2011). Role for PPAR*γ* in obesity-induced hepatic steatosis as determined by hepatocyte- and macrophage-specific conditional knockouts. *The FASEB Journal*.

[20] Inoue M., Ohtake T., Motomura W. (2005). Increased expression of PPAR*γ* in high fat diet-induced liver steatosis in mice. *Biochemical and Biophysical Research Communications*.

[21] Degrace P., Moindrot B., Mohamed I. (2006). Upregulation of liver VLDL receptor and FAT/CD36 expression in LDLR^ -/-^ apoB^100/100^ mice fed trans-10,cis-12 conjugated linoleic acid. *Journal of Lipid Research*.

[22] Lelliott C. J., Ljungberg A., Ahnmark A. (2007). Hepatic PGC-1*β* overexpression induces combined hyperlipidemia and modulates the response to PPAR*α* activation. *Arteriosclerosis, Thrombosis, and Vascular Biology*.

[23] Breslow J. L., Sloan H. R., Ferrans V. J., Anderson J. L., Levy R. I. (1973). Characterization of the mouse liver cell line FL83B. *Experimental Cell Research*.

[24] Schadinger S. E., Bucher N. L. R., Schreiber B. M., Farmer S. R. (2005). PPAR*γ*2 regulates lipogenesis and lipid accumulation in steatotic hepatocytes. *American Journal of Physiology - Endocrinology and Metabolism*.

[25] Greenspan P., Mayer E. P., Fowler S. D. (1985). Nile red: a selective fluorescent stain for intracellular lipid droplets. *Journal of Cell Biology*.

[26] Zhang L. F., Liu L. S., Chu X. M. (2014). Combined effects of a high-fat diet and chronic valproic acid treatment on hepatic steatosis and hepatotoxicity in rats. *Acta Pharmacologica Sinica*.

[27] Fan B., Ikuyama S., Gu J.-Q. (2009). Oleic acid-induced ADRP expression requires both AP-1 and PPAR response elements, and is reduced by Pycnogenol through mRNA degradation in NMuLi liver cells. *The American Journal of Physiology—Endocrinology and Metabolism*.

[28] Martins I. J., Hone E., Chi C., Seydel U., Martins R. N., Redgrave T. G. (2000). Relative roles of LDLr and LRP in the metabolism of chylomicron remnants in genetically manipulated mice. *Journal of Lipid Research*.

[29] Ibrahimi A., Abumrad N. A. (2002). Role of CD36 in membrane transport of long-chain fatty acids. *Current Opinion in Clinical Nutrition and Metabolic Care*.

[30] Coburn C. T., Hajri T., Ibrahimi A., Abumrad N. A. (2001). Role of CD36 in membrane transport and utilization of long-chain fatty acids by different tissues. *Journal of Molecular Neuroscience*.

[31] Febbraio M., Abumrad N. A., Hajjar D. P. (1999). A null mutation in murine CD36 reveals an important role in fatty acid and lipoprotein metabolism. *The Journal of Biological Chemistry*.

[32] Medina-Gomez G., Gray S. L., Yetukuri L. (2007). PPAR gamma 2 prevents lipotoxicity by controlling adipose tissue expandability and peripheral lipid metabolism. *PLoS Genetics*.

[33] Spire C., Rogue A., Brun M., Claude N., Guillouzo A. (2010). Gene expression changes induced by PPAR gamma agonists in animal and human liver. *PPAR Research*.

[34] Farmer S. R. (2005). Regulation of PPAR*γ* activity during adipogenesis. *International Journal of Obesity*.

[35] Patsouris D., Reddy J. K., Müller M., Kersten S. (2006). Peroxisome proliferator-activated receptor *α* mediates the effects of high-fat diet on hepatic gene expression. *Endocrinology*.

[36] Yu S., Matsusue K., Kashireddy P. (2003). Adipocyte-specific gene expression and adipogenic steatosis in the mouse liver due to peroxisome proliferator-activated receptor *γ*1 (PPAR*γ*1) overexpression. *Journal of Biological Chemistry*.

